# Supplementary material for: CurQ+, a Next-Generation Formulation of Curcumin, Ameliorates Growth Plate Chondrocyte Stress and Increases Limb Growth in a Mouse Model of Pseudoachondroplasia
Source: Int J Mol Sci. 2023 Feb 14;24(4):3845. doi: 10.3390/ijms24043845 (PMC9959842; doi:10.3390/ijms24043845)
Supplement: Supplementary file 1 [file ijms-24-03845-s001.zip › ijms-1965009-SM file.pdf]

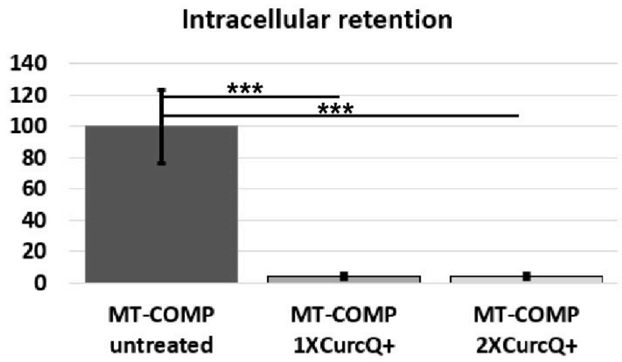

Figure S1. CurQ+ treatment dramatically reduces intracellular MT-COMP protein retention. Intracellular signal from MT-COMP untreated and 1X and 2X CurQ+ treatment was quantified using Image J. Intracellular human COMP signal from MT-COMP untreated was set to 100 and both 1X and 2X CurQ+ treatment lowered signal to approximately 4. Signal was compared using *t* test. *** = $p < 0.0005$.
